# Microbial Translocation Is Associated with Increased Monocyte Activation and Dementia in AIDS Patients

**DOI:** 10.1371/journal.pone.0002516

**Published:** 2008-06-25

**Authors:** Petronela Ancuta, Anupa Kamat, Kevin J. Kunstman, Eun-Young Kim, Patrick Autissier, Alysse Wurcel, Tauheed Zaman, David Stone, Megan Mefford, Susan Morgello, Elyse J. Singer, Steven M. Wolinsky, Dana Gabuzda

**Affiliations:** 1 Dana-Farber Cancer Institute and Harvard Medical School, Boston, Massachusetts, United States of America; 2 Northwestern University Medical School, Chicago, Illinois, United States of America; 3 Beth Israel Deaconess Center, Boston, Massachusetts, United States of America; 4 Lemuel Shattuck Hospital, Jamaica Plain, Massachusetts, United States of America; 5 Mount Sinai Medical Center, New York, New York, United States of America; 6 University of California Los Angeles Medical Center, Los Angeles, California, United States of America; New York University School of Medicine, United States of America

## Abstract

Elevated plasma lipopolysaccharide (LPS), an indicator of microbial translocation from the gut, is a likely cause of systemic immune activation in chronic HIV infection. LPS induces monocyte activation and trafficking into brain, which are key mechanisms in the pathogenesis of HIV-associated dementia (HAD). To determine whether high LPS levels are associated with increased monocyte activation and HAD, we obtained peripheral blood samples from AIDS patients and examined plasma LPS by *Limulus* amebocyte lysate (LAL) assay, peripheral blood monocytes by FACS, and soluble markers of monocyte activation by ELISA. Purified monocytes were isolated by FACS sorting, and HIV DNA and RNA levels were quantified by real time PCR. Circulating monocytes expressed high levels of the activation markers CD69 and HLA-DR, and harbored low levels of HIV compared to CD4^+^ T-cells. High plasma LPS levels were associated with increased plasma sCD14 and LPS-binding protein (LBP) levels, and low endotoxin core antibody levels. LPS levels were higher in HAD patients compared to control groups, and were associated with HAD independently of plasma viral load and CD4 counts. LPS levels were higher in AIDS patients using intravenous heroin and/or ethanol, or with Hepatitis C virus (HCV) co-infection, compared to control groups. These results suggest a role for elevated LPS levels in driving monocyte activation in AIDS, thereby contributing to the pathogenesis of HAD, and provide evidence that cofactors linked to substance abuse and HCV co-infection influence these processes.

## Introduction

Immune activation is a strong predictor of HIV disease progression [1], [2]. Elevated plasma endotoxin (bacterial lipopolysaccharide, LPS, a component of Gram-negative bacteria), a consequence of translocation of bacterial products from a leaky gut, is a likely cause of immune activation in HIV infection [3]. LPS triggers monocyte (Mo) activation via CD14 and TLR4-mediated signaling, resulting in release of soluble CD14 and pro-inflammatory cytokines. Sustained Mo stimulation leads to LPS tolerance [4]. Brenchley et al. found correlations between plasma LPS levels, Mo tolerance to LPS ex vivo, and T-cell activation markers, and proposed that T-cell activation is an indirect consequence of LPS-mediated Mo stimulation [3].

Alterations in circulating Mo linked to HIV disease progression include increased expression of pro-inflammatory cytokines and Mo activation markers [5], [6], [7]. CD16/FcγRIII expression on Mo distinguishes a minor CD16^+^ subset [8], [9] that expresses higher TNF and IL-1 [5], and higher HLA-DR, CD40, and CD86 levels compared to CD16^−^ Mo. CD16^+^ Mo represent 5–10% of circulating Mo in healthy individuals [8], but are dramatically expanded in HIV-infected patients [5], [10], particularly during progression to AIDS [6], [7]. Although Mo do not support productive HIV infection in vitro [11] and represent a minor viral reservoir in HIV-infected patients [12], [13], [14], CD16^+^ Mo may be preferentially susceptible to infection [14], [15], [16]. Furthermore, CD16^+^ Mo-derived macrophages form conjugates with T-cells that promote T-cell activation and HIV replication [17], [18]. Activated Mo play a key role in the pathogenesis of HIV-associated dementia (HAD) and minor cognitive motor disorder (MCMD) [7], [19], [20], [21], [22] by carrying virus into the CNS, supporting productive infection upon differentiation into macrophages, and producing neurotoxic factors [7], [21]. An increased frequency of CD16^+^/CD69^+^ activated Mo [5] was associated with HAD in the pre-HAART era [6], [7]. HAART decreases the frequency of CD16^+^ Mo [10], reduces HIV levels in brain, and improves neurocognitive function [19], but neurocognitive impairment (NCI) still occurs in ∼10–20% of AIDS patients [19], [20], [21]. Host factors that drive Mo activation in patients who develop HAD have not been defined.

Here we investigated the relationship between plasma LPS, alterations in circulating Mo, and HAD in AIDS patients. High plasma LPS and LPS-binding protein (LBP) levels, together with low endotoxin core antibody (EndoCAb) levels, were associated with increased soluble markers of Mo activation and HAD. Plasma LPS levels were higher in AIDS subjects with IV heroin or ethanol abuse, or co-infection with Hepatitis C virus (HCV) compared to control groups. These findings suggest a role for elevated LPS levels in triggering Mo activation during HIV infection, thereby contributing to HAD pathogenesis, and identify cofactors that may influence these processes.

## Materials and Methods

### Subjects

AIDS patients with CD4 counts <300 cells/µl were recruited at the Lemuel Shattuck Hospital (n = 49) or at 4 sites in the National NeuroAIDS Tissue Consortium (NNTC) (Manhattan HIV Brain Bank, National Neurological AIDS Bank, California NeuroAIDS Tissue Network, Texas NeuroAIDS Research Center) (n = 70) with written informed consent and IRB approval. HIV-/HCV-uninfected blood was from Research Blood Components (Brighton, MA). Shattuck Hospital subjects were recruited based on presence or absence of an HAD diagnosis. NNTC subjects were selected by database searches based on patterns of substance abuse (determined by PRISM (psychiatric research interview for substance and mental disorders) diagnoses and urine tox screens) irrespective of HAD or other forms of NCI. HAD or MCMD diagnoses were determined using American Academy of Neurology diagnostic criteria [19], [23] and asymptomatic neurocognitive impairment (ANI) diagnosis was determined using HIV Neurobehavioral Research Center (HNRC) criteria [24] based on formal neurocognitive testing and/or neurological evaluation and lack of other known cause for NCI. Patients with severe psychiatric diagnoses that might confound neurological evaluation, a confounding neurological disorder (i.e., CNS opportunistic infection, brain neoplasm), or active bacterial or opportunistic infection were excluded.

### Antibodies

Fluorochrome-conjugated Abs used for FACS analysis were CD14, CD16, CD19, CD33, CD16b, CD66b, CD56, and CD3 (Beckman Coulter); CD4, CCR5, CD69, and HLA-DR (BD Pharmingen).

### Cell sorting

PBMC were isolated from peripheral blood by Ficoll-Paque centrifugation [17], [25]. Mo were isolated by negative selection using immunobeads (Miltenyi) [17], [25] or by FACS sorting. CD3^+^ T-cells were isolated using CD3 immunobeads (Miltenyi), and CD4^+^ T-cells were sorted by FACS. PBMC depleted of CD3^+^ T-cells were stained with PE-CD14 and PE-Cy5 CD16 Abs and FITC-conjugated Abs against T-cell (CD3), granulocyte (CD16b/CD66b), B cell (CD19), NK cell (CD56), and dendritic cell (CD1c) markers. Sorted T-cells and Mo were >98% pure as determined by FACS.

### Quantification of HIV DNA and RNA

HIV DNA and RNA in CD4^+^ T-cells and Mo were quantified by real time PCR and RT-PCR (sensitive to 50 copies). Total DNA and RNA was isolated using Qiagen DNA-EZ and RNA-EZ isolation kits. DNA from 10^5^ cells was amplified for 45 cycles using Taqman Universal PCR Master Mix (Applied Biosystems) and HIV Gag primers and probe [26]. Sequential dilutions of 8E5 cells were used to quantify Gag and CCR5 copy number. For qRT-PCR, RNA from 10^5^ cells was reverse transcribed using the High Capacity cDNA Archive kit (Applied Biosystems) and amplified with HIV Gag primers and probes [26].

### Isolation of replication-competent HIV

CD4^+^ T-cells and Mo isolated from HIV-infected patients were cultured with Mo:CD4^+^ T-cell co-cultures from a HIV-uninfected donor pre-stimulated with PHA/IL-2 as described, a method that isolates both CCR5 and CXCR4-using HIV strains efficiently [17], [18], [27]. HIV p24 was quantified in culture supernatants by ELISA (Perkin Elmer).

### Quantification of plasma LPS, lipopolysaccaride binding-protein, IgM endotoxin core antibody, soluble CD14, IL-6, and CCL2

Plasma LPS levels were quantified using the Diazo-coupled *Limulus* amebocyte lysate (LAL) assay (Associates of Cape Cod Inc.) according to manufacturer's protocol. Briefly, samples diluted 1/5 were inactivated for 30 min at 65°C and incubated with LAL for 30 min at 37°C. Addition of reagents led to formation of a magenta derivative that absorbs light at 570 nm. Plasma soluble CD14 (sCD14), IL-6, and CCL2 levels were quantified by ELISA (R&D Systems). Lipopolysaccharide binding-protein (LBP) and IgM endotoxin core antibody (EndoCAb) levels in plasma were quantified by ELISA (Cell Sciences).

## Results

### Increased plasma LPS and sCD14 are associated with HAD

**Figure 1:**
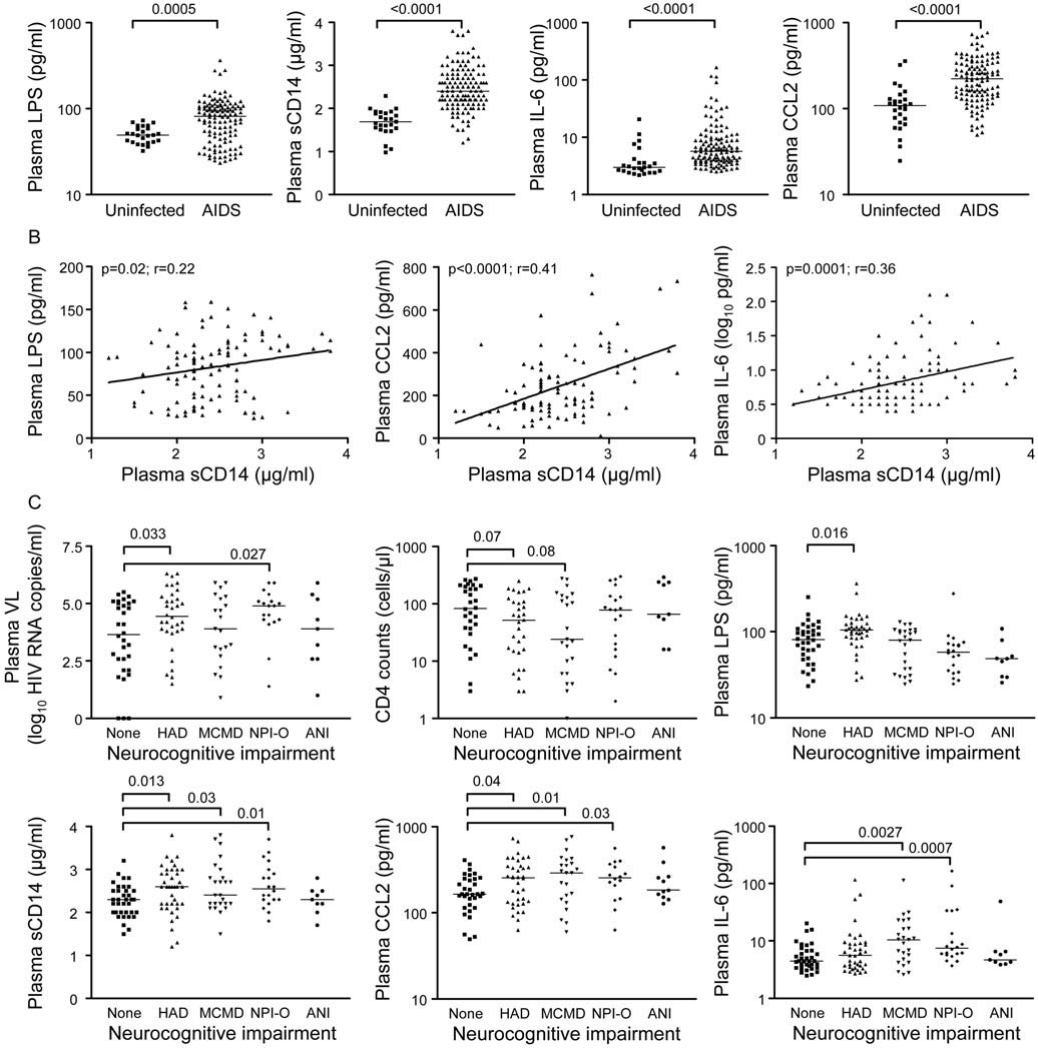
Increased plasma LPS, sCD14, and CCL2 are associated with HAD. (A) Levels of LPS, sCD14, CCL2, and IL-6 were quantified in plasma of AIDS patients (n = 119) and uninfected controls (n = 25). (B) Spearman correlation (r and p-values) was calculated to determine the relationship between the LPS levels and sCD14, and between sCD14 and plasma CCL2 or IL-6. (C) Levels of plasma VL, CD4 counts, LPS, sCD14, CCL2, and IL-6 were compared in AIDS patients classified in 5 groups based on the degree of neurocognitive impairment (NCI) (None, No NCI; HAD, HIV-associated dementia; MCMD, minor cognitive and motor disorder; NPI-O, neuropsychiatric impairment due to conditions other than HIV; and ANI, asymptomatic NCI). Kruskal-Wallis test determined significant differences between the 5 groups in 1C. Median values in 1A and 1C are indicated as horizontal lines and significant differences between the 4 NCI groups *versus* the no NCI group (None) were determined using the Mann-Whitney test.

To investigate the potential role of circulating LPS in triggering Mo activation, plasma LPS levels together with soluble markers of Mo activation (i.e., sCD14 [3], IL-6, and CCL2 [28]) were compared between AIDS patients classified according to NCI diagnoses into 5 groups (No NCI, HAD, MCMD, neuropsychiatric impairment due to cause other than HIV (NPI-O), and ANI). The cohort consisted of 119 AIDS patients enrolled at the Shattuck Hospital (n = 49) or NNTC (n = 70) (Table 1), and had relatively high plasma VL and low CD4 counts compared to other current cohorts, together with a high frequency of intravenous drug abuse (IVDU). 61 of 94 subjects were HAART failures (>1,000 HIV RNA copies/ml), 11 were on HAART <8 weeks, and 14 were not on HAART. Plasma levels of LPS, sCD14, IL-6, and CCL2 were higher in AIDS compared to uninfected subjects (Figure 1A). Plasma LPS levels correlated positively with sCD14 (Figure 1B), but not CCL2 or IL-6. sCD14 levels correlated positively with CCL2, IL-6 (Figure 1B), and plasma VL (p = 0.03; r = 0.19) and negatively with CD4 counts (p = 0.0012, r = −0.29). Significant differences in plasma VL, LPS, sCD14, IL-6, and CCL2 but not CD4 counts, were observed between the NCI groups (Kruskal-Wallis, p <0.05). Plasma VL was higher in HAD and NPI-O subjects compared to those with no NCI. CD4 counts trended toward lower levels in HAD or MCMD subjects compared to those with no NCI (Figure 1C). Compared to subjects with no NCI, LPS levels were higher in HAD, sCD14 and CCL2 levels were higher in HAD, MCMD, and NPI-O, and IL-6 levels were higher in MCMD and NPI-O subjects (Figure 1C). In patients with CD4 counts <100, LPS and sCD14 levels were also higher in HAD subjects (median, 106.5 pg/ml LPS and 2.65 µg/ml sCD14) compared to those with no NCI (median, 68.1 pg/ml LPS and 2.45 µg/ml sCD14; p = 0.0079 and p = 0.024, respectively).

**Table 1:** Demographic and clinical characteristics of AIDS patients in the study cohort.

**Gender**	Male	n = 89
	Female	n = 30
**Race**	Hispanic	n = 25
	Caucasian	n = 48
	African American	n = 43
	Other	n = 3
**HAART**	≥8 weeks	n = 94
	<8 weeks	n = 11
	None	n = 14
**Risk Factor**	Sexual transmission	n = 66
	Intravenous drug use	n = 53
**Neurocognitive impairment (NCI)**	No NCI	n = 32
	HAD	n = 28
	MCMD	n = 25
	NPI-O	n = 20
	ANI	n = 9
	Unable to assign	n = 5
**Age (years)**	Mean±SD	45±8
	Median (range)	44 (25–69)
**Plasma HIV RNA (copies/ml)**	Mean±SD	150,600±330,100
	Median (range)	19,940 (<50–2,210,000)
**CD4 T-cell count (cells/µl)**	Mean±SD	110±197
	Median (range)	64 (1–299)

HAART, highly active antiretroviral therapy; NCI, neurocognitive impairment; HAD, HIV-associated dementia, MCMD, minor cognitive and motor disorder; NPI-O, neuropsychiatric impairment due to conditions other than HIV; ANI, asymptomatic NCI.

High LPS or sCD14 levels were associated with an increased frequency of HAD, but not MCMD, NPI-O, or ANI (Tables 2 and 3). Multivariate analysis in logistic regression models demonstrated that LPS levels were a predictor of HAD independently of plasma VL and CD4 counts, whereas sCD14 levels were a predictor of HAD independently of plasma VL and LPS, but in relationship with CD4 counts and plasma levels of CCL2 and IL-6 (p<0.05).

**Table 2:** Distribution of patients with HAD and without neurocognitive impairment (NCI) stratified by plasma LPS levels.

Group A–high plasma LPS		Group B-low plasma LPS	
No NCI	HAD	No NCI	HAD
n = 18	n = 31	n = 18	n = 8

AIDS patients with CD4 counts <300 cells/µl were classified based on plasma LPS levels at one (n = 41) or two (n = 17) visits into 2 groups (cutoff 79 pg/ml, the median LPS value in the AIDS cohort). The distribution of patients with HAD or without NCI in these 2 groups was analyzed for statistical significance using the Chi-square test (p = 0.007) and odds ratio (3.8, with 95% confidence interval 1.403–10.7) with Prism4 software. The distribution of patients with MCMD and without NCI in the two groups was similar (p = 0.8).

**Table 3:** Distribution of patients with HAD and without neurocognitive impairment (NCI) stratified by plasma sCD14 levels.

Group A–high plasma sCD14		Group B-low plasma sCD14	
No NCI	HAD	No NCI	HAD
n = 12	n = 23	n = 24	n = 16

AIDS patients with CD4 counts <300 cells/µl were classified based on plasma sCD14 levels at one (n = 41) or two (n = 17) visits into 2 groups (cutoff 2.4 µg/ml, the median sCD14 value in the AIDS cohort). The distribution of patients with HAD or without NCI in these 2 groups was analyzed for statistical significance using the Chi-square test (p = 0.026) and odds ratio (2.8, with 95% confidence interval 1.121–7.376). The distribution of MCMD patients in the two groups was similar (p = 0.2).

### Substance abuse and HCV coinfection are associated with high plasma LPS

**Figure 2:**
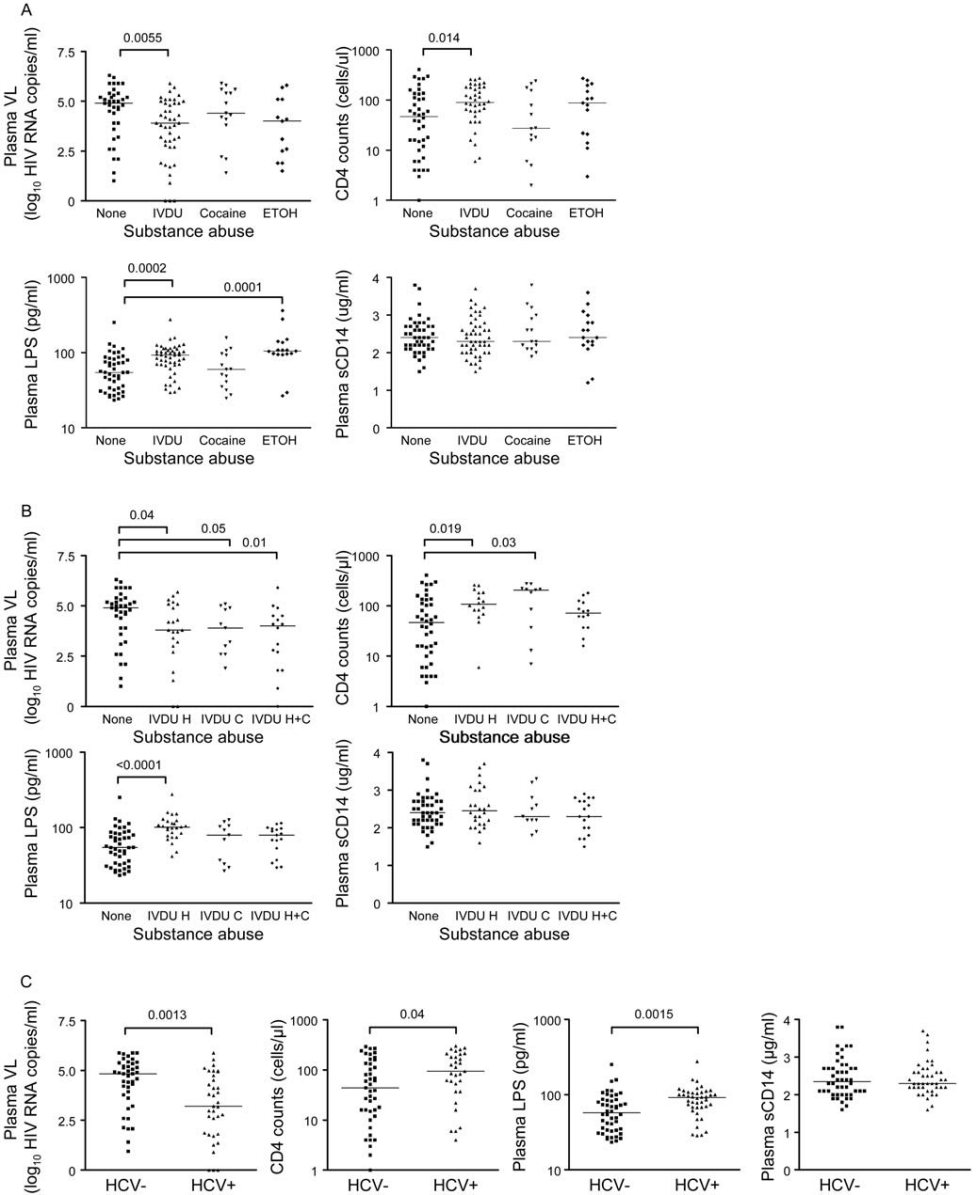
Substance abuse and HCV co-infection are associated with high plasma LPS levels. (A) CD4 counts and plasma levels of VL, LPS, and sCD14 were compared in AIDS patients with or without substance abuse as indicated. (B) CD4 counts and plasma levels of VL, LPS, and sCD14 were compared in AIDS patients with IVDU heroin (H), cocaine (C), and H+C. (C) CD4 counts and plasma levels of VL, LPS, and sCD14 were compared in AIDS patients with and without HCV co-infection. Median values are indicated as horizontal lines and statistical significance between groups was calculated using the Mann-Whitney test; significant differences (p<0.05) are indicated.

An increased incidence of bacterial infections occurs in IVDU [29], [30]. Whether LPS levels are influenced by substance abuse in AIDS patients is unknown. The study cohort included many subjects with active IVDU or other types of substance abuse. We therefore examined the relationship between specific patterns of substance abuse and plasma LPS or sCD14 levels. Patients were classified into 4 groups based on patterns of substance abuse: no substance abuse, IVDU (H, heroin, C, cocaine, and H+C, both heroin and cocaine), non-intravenous cocaine (i.e., via mucosal routes), and ethanol. Significant differences in plasma VL, LPS, and CD4 counts, but not sCD14 levels, were observed between groups (Figure 2A–B) (Kruskal-Wallis, p<0.05). IVDU, but not non-intravenous cocaine abuse, was associated with higher plasma LPS levels, while plasma VL and CD4 counts were lower and higher, respectively, in IVDU subjects compared to those with no substance abuse (Figure 2A). Ethanol abuse was associated with higher plasma LPS levels but no significant differences in plasma VL or CD4 counts compared to the control group (Figure 2A). IVDU heroin users, but not IVDU heroin/cocaine or cocaine users, had higher plasma LPS levels despite lower plasma VL and higher CD4 counts compared to subjects with no substance abuse (Figure 2B). Thus, IV heroin and ethanol abuse are associated with increased plasma LPS levels in AIDS patients.

The liver plays a critical role in the response to infections and LPS clearance [31], [32]. HCV co-infection is frequent in IVDU, and has been associated with an increased incidence of NCI in AIDS patients [33], [34], [35]. Plasma LPS, but not sCD14 levels, were higher in AIDS patients co-infected with HCV compared to HCV- subjects, even though plasma VL was lower and CD4 counts were higher in HCV+ compared to HCV- subjects (Figure 2C). Among HCV+ subjects, LPS levels were higher in IVDU compared to subjects with remote or no substance abuse (93.8 *versus* 39.5 pg/ml LPS, median, n = 34 and n = 6, respectively, p = 0.009). Thus, HCV and IVDU are independently associated with increased LPS in AIDS patients.

### Increased LBP and decreased EndoCAb levels are associated with HAD

**Figure 3:**
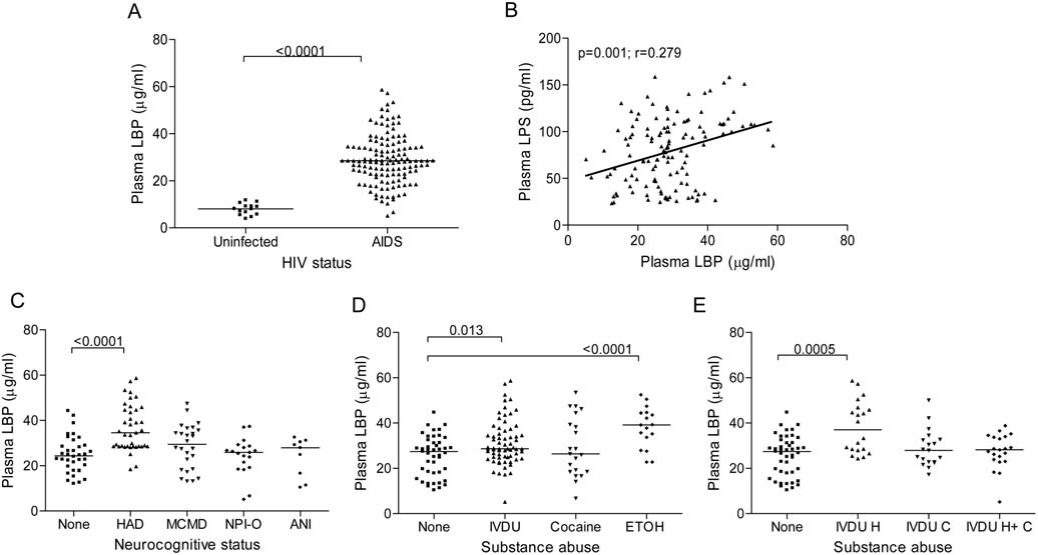
Increased plasma lipopolysaccharide binding protein (LBP) levels in AIDS patients with HAD. (A) LBP levels were quantified in plasma from AIDS patients (n = 119) and uninfected controls (n = 14) by ELISA. (B) Spearman correlation (r and p-values) was calculated to determine relationships between LBP levels and plasma LPS. (C) LBP levels were compared between AIDS patients classified into 5 groups based on the level of NCI as in Figure 1C or (D–E) classified into groups based on patterns of substance abuse as in Figure 2A–B. Median values are indicated as horizontal lines and statistical significance between groups was calculated using the Mann-Whitney test; significant differences (p<0.05) are indicated.

**Figure 4:**
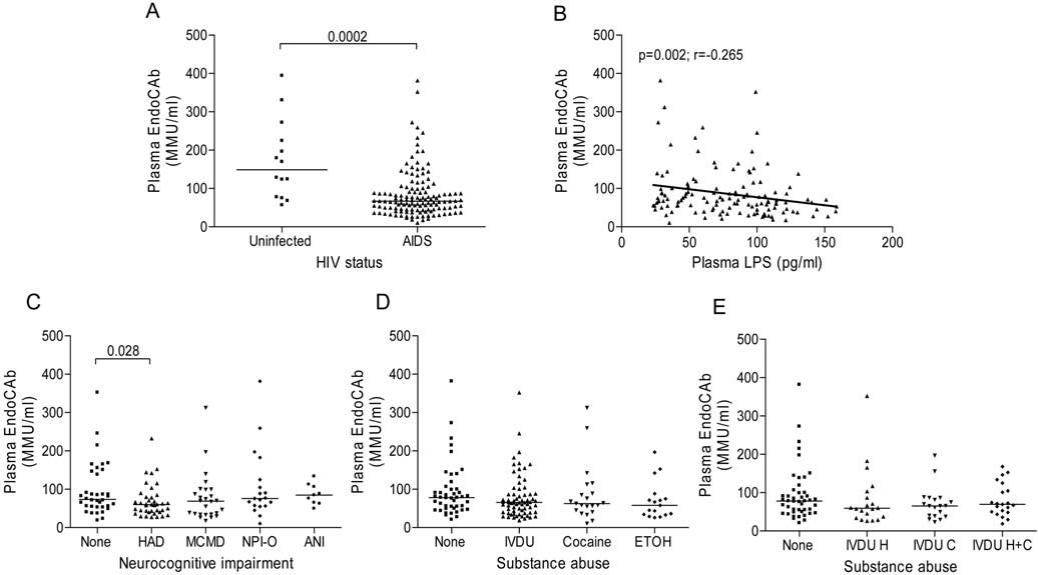
Decreased plasma IgM endotoxin core antibody (EndoCAb) levels in AIDS patients with HAD. (A) Levels of IgM EndoCAb were quantified in plasma from AIDS patients (n = 119) and uninfected controls (n = 14) by ELISA. (B) Spearman correlation (r and p-values) was calculated to determine relationships between IgM EndoCAb levels and plasma LPS. (C) IgM EndoCAb levels were compared between AIDS patients classified into 5 groups based on the level of NCI as in Figure 1C or (D–E) classified into groups based on patterns of substance abuse as in Figure 2A–B. Median values are indicated as horizontal lines and statistical significance between groups was calculated using the Mann-Whitney test; significant differences (p<0.05) are indicated.

Increased LPS in the circulation promotes hepatic synthesis of LBP, a plasma protein that increases the binding of LPS to CD14. In some clinical studies, plasma LBP levels reflected long-term exposure to circulating endotoxin more accurately than plasma endotoxin levels [36], [37]. Microbial translocation in HIV-infected patients is associated with increased LBP levels and decreased IgM antibodies to the LPS core oligosaccharide (EndoCAb) [3]. We measured plasma levels of LBP and EndoCAb in AIDS patients with and without NCI and uninfected controls, and found that LBP levels were significantly higher in AIDS patients compared to uninfected subjects (Figure 3A) and correlated positively with plasma LPS (Figure 3B). Plasma LBP levels were significantly higher in HAD subjects compared to those with no NCI (Figure 3C). Among subjects classified according to different patterns of substance abuse, LBP levels were higher in subjects with active heroin IVDU or ethanol abuse compared to those with no substance abuse (Figures 3D–E). Plasma IgM EndoCAb levels were significantly lower in AIDS compared to uninfected subjects (Figure 4A) and correlated negatively with plasma LPS levels (Figures 4B). Plasma EndoCAb levels were slightly lower in AIDS patients with HAD compared to those with no NCI (Figure 4C). Plasma EndoCAb levels were not significantly different among subject groups classified according to different patterns of substance abuse (Figure 4E–F). Thus, LBP levels are higher in HAD subjects compared to those with no NCI, and in subjects with IV heroin or ethanol abuse compared to those with no substance abuse.

### Increased CD16^+^ monocyte frequency is associated with AIDS but not with HAD

**Figure 5:**
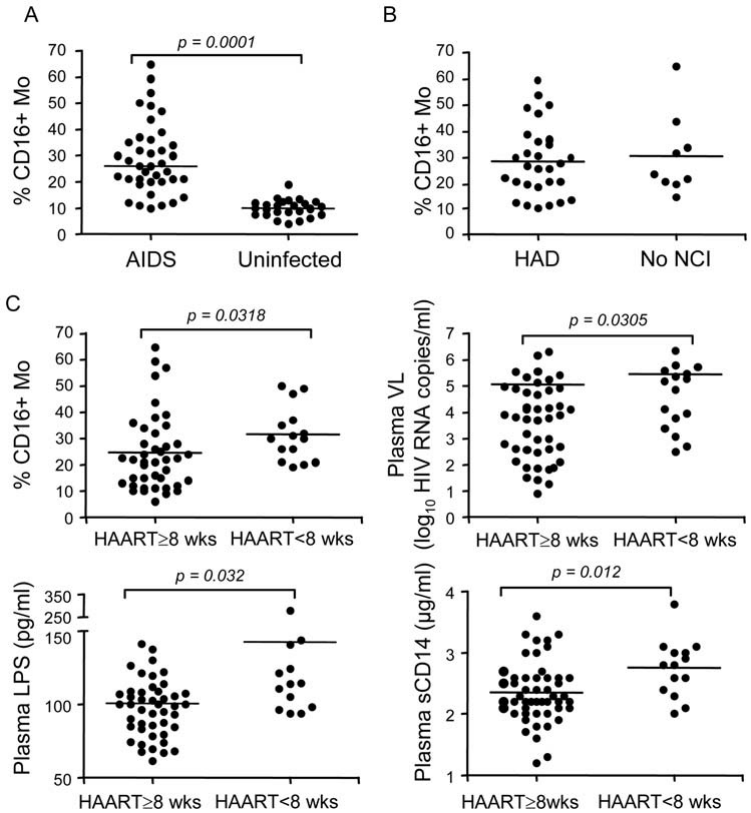
Increased CD16^+^ monocyte frequency in AIDS patients with and without HAD. (A) The frequency of CD16^+^ Mo within the total Mo population was analyzed in AIDS (*n = 38*) and uninfected subjects (*n = 25*). Total and CD16^+^ Mo were distinguished from granulocytes by HLA-DR and lack of CD16b/CD66b expression, and from NK cells by higher forward and side scatter characteristics (FSC and SSC), CD14, CD4, and CD33, and lack of CD56 expression [25]. (B) The frequency of CD16^+^ Mo was compared in AIDS patients with HAD (n = 29) and no NCI (n = 9). (C) The frequency of CD16^+^ Mo, plasma VL, and plasma levels of LPS and sCD14 were compared in patients receiving HAART ≥8 weeks (wks) and untreated or on HAART <8 wks. Median values are indicated as horizontal lines and statistical significance between groups was calculated using the Mann-Whitney test; significant differences (p<0.05) are indicated.

In the pre-HAART era, preferential expansion of the CD16^+^ Mo subset was associated with HAD a study by Pulliam et al. [6]. We therefore examined whether the CD16^+^ Mo subset is preferentially expanded in HAD subjects in the present study cohort, and whether high LPS levels were associated with preferential expansion of this Mo subset. In AIDS patients recruited at the Shattuck Hospital (n = 49), the frequency of Mo expressing CD16 was significantly increased compared to uninfected controls (Figure 5A), whereas absolute Mo counts were similar in AIDS (380±166 cells/µl, n = 37) and control subjects (435±220 cells/µl, n = 14; p = 0.442) based on analysis of white blood cell (WBC) counts. High plasma VL (cutoff 10,000 HIV RNA copies/ml) was associated with an increased frequency of CD16^+^ Mo (median 20.5% versus 29%; p = 0.01), together with decreased CD4 counts (median 109 versus 27; p = 0.006). The frequency of CD16^+^ Mo was high in both HAD and no NCI subjects compared to uninfected controls, with no significant difference between the HAD and no NCI groups (Figure 5B). Plasma VL and CD4 counts were significantly increased (p = 0.025) and decreased (p = 0.0009), respectively, in HAD compared to no NCI subjects. Stratification of subjects according to high versus low frequency of CD16^+^ Mo (cutoff 22.5%, median) demonstrated that CCL2 levels were slightly higher in subjects with a high frequency of CD16^+^ Mo (median 263 *versus* 165 pg/ml, p = 0.09), while LPS, sCD14, and IL-6 levels were not significantly different. Thus, the CD16^+^ Mo subset is expanded in patients with AIDS, but expansion of this Mo subset is not significantly different between HAD subjects and those with no NCI in this study cohort.

Immune restoration in patients on HAART increases the likelihood of neurocognitive recovery [38]. In addition to decreased plasma VL and increased CD4 counts, patients on HAART ≥8 weeks had a lower frequency of CD16^+^ Mo, lower LPS, and lower sCD14 levels compared to those on HAART <8 weeks or not on HAART (Figure 5C). Comparison of LPS levels in 12 AIDS patients between two consecutive visits revealed a significant decrease in LPS attributable to HAART (≥8 wks) (100.8 *versus* 84.7 pg/ml LPS, median, p = 0.04), together with a reduction in plasma VL (69,250 *vs.* 3,424 HIV RNA copies/ml, median, p = 0.04).

**Figure 6:**
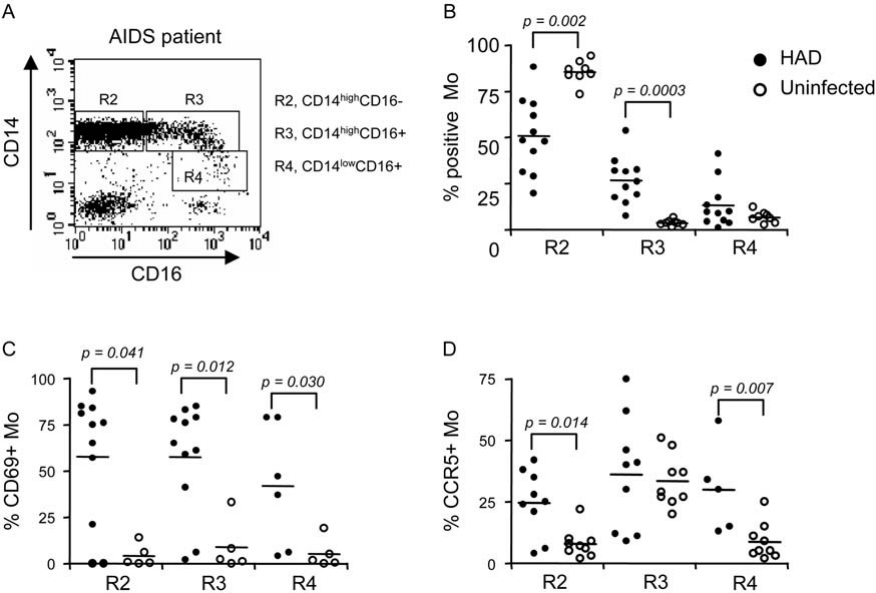
Upregulation of CD69 and CCR5 expression on CD16^+^ and CD16^−^ monocyte subsets. (A) PBMC from AIDS subjects were stained with fluorochrome-conjugated Abs. CD14 and CD16 expression identified three Mo subsets: CD14^high^CD16^−^ (gate R2), CD14^high^CD16^+^ (gate R3), and CD14^low^CD16^+^ (gate R4). (B) The frequency of each Mo subset was compared in AIDS (*n = 11*) and uninfected subjects (*n = 8*). The expression of CD69 (C) and CCR5 (D) was analyzed for each Mo subset from AIDS (*n = 9–11*) and uninfected subjects (*n = 5–9*). Median values are indicated as horizontal lines, and statistical significance in 6B–D was calculated using the Mann-Whitney test.

Mo from AIDS patients with HAD were analyzed for expression of the activation markers HLA-DR, CD69, and the HIV co-receptor CCR5. HLA-DR was expressed on >95% of Mo from both HAD and control subjects but the MFI was higher in HAD compared to control subjects (165±69 *vs.* 66±53, n = 5–7, p = 0.03). The level of CD14 expression identified two CD16^+^ Mo subsets: CD14^high^CD16^+^ and CD14^low^CD16^+^ [9], [25], with preferential expansion of the CD14^high^CD16^+^ Mo subset in HAD subjects (Figure 6A–B). CD69 expression was upregulated on all three Mo subsets, whereas CCR5 expression was increased on CD14^high^CD16^−^ and CD14^low^CD16^+^ Mo from HAD compared to control subjects (Figure 6C–D). These results indicate preferential expansion of CD14^high^CD16^+^ Mo and activation of all three Mo subsets in HAD patients.

### Monocytes harbor low levels of HIV compared to CD4^+^ T-cells

**Figure 7:**
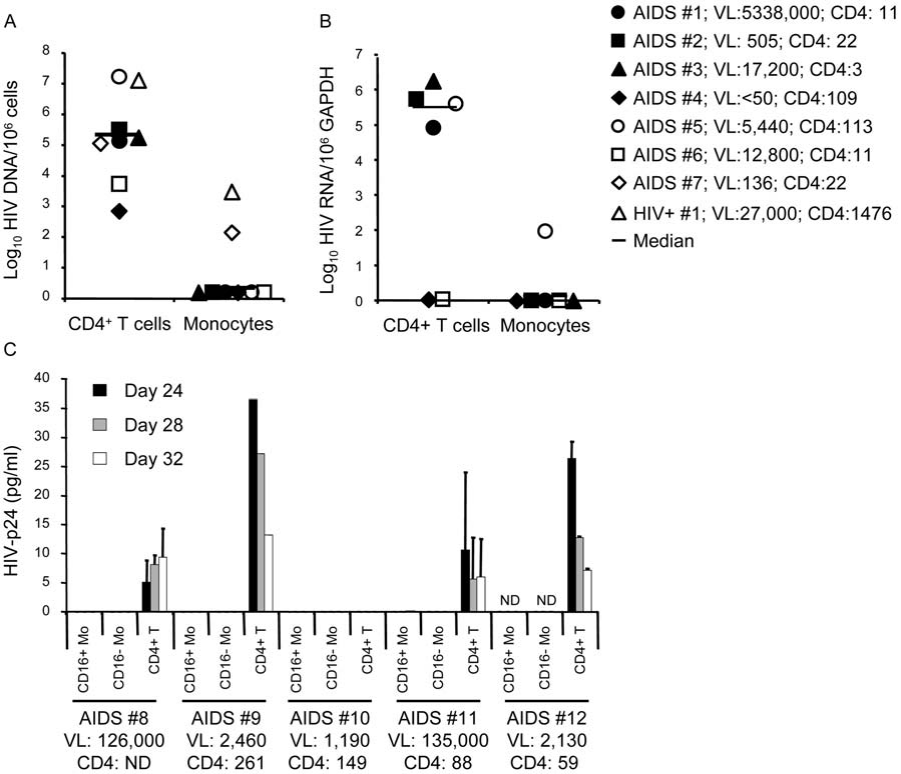
Monocytes are a minor reservoir for HIV replication compared to CD4+ T-cells *in vivo*. Highly pure CD4^+^ T-cells and total or CD16^+^ and CD16^−^ Mo subsets were sorted from the same donor peripheral blood sample by FACS from HIV-infected patients with (*n = 12*) or without (*n = 1*) AIDS. 8 of the 12 AIDS subjects had HAD. Levels of cell-associated (A) HIV DNA and (B) RNA were quantified by real time PCR and RT-PCR, respectively. (C) CD4^+^ T-cells and CD16^+^ or CD16^−^ Mo (10^5^ cells/well) from patients with high plasma VL were co-cultured with PHA/IL-2 activated Mo∶CD4^+^ T-cell co-cultures from HIV-uninfected subjects (Mo∶T-cell ratio of 1∶2; 0.5×10^6^ cells/well). Co-cultures were maintained up to 32 days and supernatants were recovered every 4 days. Shown are levels of HIV-p24 in supernatants quantified by ELISA at days 24, 28, and 32.

To quantify HIV DNA and RNA levels in Mo and CD4^+^ T-cells isolated from peripheral blood, we used real time PCR/RT-PCR. Relatively high levels of HIV DNA and RNA were detected in CD4^+^ T-cells from 8 of 8 and 4 of 6 AIDS patients, respectively, whereas levels were below the limit of detection in Mo from 6 of 8 and 5 of 6 patients, respectively (Figure 7A–B). HIV DNA and RNA levels in Mo were significantly lower compared to that in CD4^+^ T-cells from matched donors (Figure 7A–B). Replication-competent HIV was isolated from CD4^+^ T-cells, but not from CD16^+^ or CD16^−^ Mo, in 4 of 5 donors (Figure 7C). Thus, circulating Mo are a minor reservoir for HIV compared to CD4^+^ T-cells in AIDS patients.

## Discussion

In this study, we investigated the association between markers of microbial translocation, alterations in circulating monocytes, and neurocognitive impairment (NCI) in a cohort of AIDS patients with a high frequency of IVDU and HCV co-infections and relatively high plasma VL. High LPS and LBP together with decreased EndoCAb levels were associated with increased sCD14 levels and HIV-associated dementia (HAD). LPS levels were higher in AIDS patients with IV heroin or ethanol abuse, or with HCV co-infection, and were lower in patients on HAART, compared to control groups. These findings support the idea that increased circulating LPS, a consequence of microbial translocation, contributes to Mo activation in chronic HIV infection/AIDS, as proposed by Brenchley et al [1], [3], and provide evidence that circulating LPS may be linked to development of HAD via increased trafficking of activated Mo into brain [7], [20], [21], [22]. Our results also suggest that cofactors linked to IV heroin and/or ethanol abuse and HCV co-infection may influence these processes.

Plasma LPS levels are dramatically increased in certain pathological conditions that include sepsis, inflammatory bowel disease (IBD), graft versus host disease (GVHD), and HIV infection [1], [3]. The gastrointestinal tract is a major reservoir for both commensal and pathogenic bacteria, in addition to being a major site for HIV replication [1]. The level of microbial translocation was previously correlated with immune activation and CD4^+^ T cell depletion in HIV-infected patients [1], [3], [39]. Here, we demonstrate that high plasma LPS levels, together with increased Mo activation markers (i.e., sCD14, CCL2, and IL-6), are associated with HAD and/or minor cognitive and motor disorder (MCMD). LPS levels predicted HAD independently of VL and CD4 counts, whereas sCD14 levels predicted HAD independently of VL but in relationship with CD4 counts and plasma IL-6 and CCL2 levels. We also found an association between high VL or low CD4 counts and HAD, consistent with previous reports [20], [21], indicating that both virologic and immune control in the periphery are linked to key events involved in HIV neuropathogenesis.

LBP, which is produced in the liver during the acute phase of infections, binds to LPS and triggers CD14-dependent Mo activation [40]. Plasma LBP and LPS levels both have prognostic significance in patients with sepsis [37]. Plasma LBP levels are increased in HIV-infected patients and correlate positively with the degree of microbial translocation [3]. Consistent with these results, we found a direct correlation between LPS and LBP levels in AIDS patients. In addition, we demonstrated that increased LBP levels are associated with HAD. Plasma levels of immunoglobulin-M (IgM) antibodies to the LPS core oligosaccharide (EndoCAb), which are normally present in human plasma and potently inhibit LPS activity [41], were significantly decreased in AIDS patients, consistent with the study by Brenchley et al. [3], probably a consequence of altered B cell function in HIV-infected individuals [42], [43]. EndoCAb levels were slightly lower in HAD subjects compared to those with no NCI, consistent with a previous study which found that HAD patients contain lower levels of anti-HIV neutralizing antibodies in plasma compared to non-HAD patients [44]. Together, these results support the idea that translocation of bacterial products from the gut or other mucosal sites in AIDS patients contributes to events involved in the development of HAD.

AIDS subjects with IV heroin or ethanol abuse had higher LPS and LBP levels compared to those with no substance abuse. Plasma LPS levels were previously reported to be increased in ethanol abusers and in HCV infection as a consequence of increased gut permeability and/or decreased hepatic function [45], [31], [32], [46]. Possible reasons for high plasma LPS in IV heroin users include increased susceptibility to bacterial infections [29], [30], high frequency of HCV co-infection, and reduced immune responses [30], [47]. Studies on the relationship between substance abuse and HIV disease progression reached different conclusions, probably due to different study designs and methodological limitations [29], [30], [48]. Our results together with previous reports that found a higher incidence or more rapid progression of HAD in IVDU [49], [50], [51] suggest that high levels of LPS may contribute to an increased frequency of NCI in heroin IVDU compared to non-IVDU AIDS patients. LPS levels were higher in patients with HCV coinfection than in HCV-negative subjects, consistent with previous studies that found endotoxemia during HCV infection [32], [46]. Thus, circulating LPS may contribute to the increased incidence of NCI that has been observed in association with HIV/HCV co-infection [33], [34], [35]. Surprisingly, sCD14 levels were not increased in AIDS patients with substance abuse or HCV coinfection; however, these subjects had higher CD4 counts and lower plasma VL compared to control groups, indicating a less advanced stage of HIV disease. Mo sensitivity to LPS stimulation is influenced by host factors that include receptor expression levels (e.g., CD14 and TLR4) and intracellular signaling pathways that control the balance between pro- (e.g., IRAK-4) [52] and anti-inflammatory responses (e.g., IRAK-M) [53]. Moreover, cytokines such as M-CSF, IFN-γ, and CCL2, which are increased in blood and other tissues during HIV/AIDS disease progression, can enhance monocyte sensitivity to LPS stimulation [32], [54], [55], [56]. Host factors and differences in disease stage may therefore explain, at least in part, why increased LPS was associated with higher sCD14 in subjects with HAD but not substance abuse or HCV coinfection compared to control groups.

In contrast to a study in the pre-HAART era [6], we found a similar high frequency of circulating CD16^+^ Mo in HAD and non-HAD patients. These different results may relate to HAART [57], phenotypic analysis of PBMC versus whole blood [6], or the high frequency of IVDU in our cohort. We found an association between an increased frequency of CD16^+^ Mo and elevated plasma CCL2, which may be relevant for neuropathogenesis, since high CCL2 levels have been associated with an increased risk of developing HAD [58] and CD16^+^ Mo produce CCL2 [28]. CD16^+^ Mo may contribute to HAD via trafficking into brain and differentiating into activated perivascular macrophages [25], which may promote CNS injury via production of CCL2, IL-6, and MMP-9 [28], and other mechanisms [7], [20], [21], [22].

Previous studies linked Mo activation [6], [7], [59] and increased plasma sCD14 [60] and CCL2 levels [58] to an increased risk of developing HAD. We found that HLA-DR and CD69 were upregulated on all Mo subsets in HAD subjects, indicating a general state of Mo activation. CCR5 was also upregulated, which may increase Mo susceptibility to HIV infection and trafficking into tissues [14]. We found an association between HAD and increased plasma sCD14 and CCL2, which are soluble markers of Mo activation [58], [60], [61]. LPS levels correlated positively with sCD14 but not CCL2 levels, suggesting that LPS triggers Mo release of sCD14 [3], while CCL2 may be induced by stimuli other than LPS [28]. LPS stimulates leukocyte recruitment into the CNS [62], and may also increase blood-brain barrier (BBB) permeability [63]. Thus, circulating LPS may contribute to HAD pathogenesis by several mechanisms.

HIV DNA and RNA were low in circulating Mo compared to CD4^+^ T-cells, suggesting that Mo represent a minor site for HIV replication compared to CD4^+^ T-cells in AIDS patients. Some studies reported that circulating Mo, particularly the CD16^+^ Mo subset [15], [16], harbor HIV DNA in some AIDS patients [13], [14], while others found little or no detectable HIV DNA in circulating Mo [12], [64]. Possible reasons for these different results include differences in Mo isolation techniques, frequency of contaminating CD4^+^ T-cells, and sensitivity of HIV detection assays. Our results are consistent with previous studies indicating that Mo contribute to HIV replication *in vivo* primarily by promoting T-cell activation and differentiating into tissue macrophages that are highly susceptible to infection [17], [18].

Our results argue in favor of a link between microbial translocation and systemic immune activation [1], [3], and suggest that LPS-induced Mo activation is associated with HIV disease progression. We provide evidence that high LPS and LBP levels, together with increased levels of Mo activation, represent interrelated risk factors for HAD. Accordingly, the balance between pro- and anti-inflammatory TLR signaling pathways triggered in response to bacterial products may influence the development of HAD. New approaches to downmodulate LPS-induced Mo activation may provide new strategies for anti-HIV and HAD therapy.

## References

[1] Brenchley JM, Price DA, Douek DC (2006). HIV disease: fallout from a mucosal catastrophe?. Nat Immunol.

[2] Grossman Z, Meier-Schellersheim M, Paul WE, Picker LJ (2006). Pathogenesis of HIV infection: what the virus spares is as important as what it destroys.. Nat Med.

[3] Brenchley JM, Price DA, Schacker TW, Asher TE, Silvestri G (2006). Microbial translocation is a cause of systemic immune activation in chronic HIV infection.. Nat Med.

[4] Cavaillon JM, Adrie C, Fitting C, Adib-Conquy M (2003). Endotoxin tolerance: is there a clinical relevance?. J Endotoxin Res.

[5] Thieblemont N, Weiss L, Sadeghi HM, Estcourt C, Haeffner-Cavaillon N (1995). CD14lowCD16high: a cytokine-producing monocyte subset which expands during human immunodeficiency virus infection.. Eur J Immunol.

[6] Pulliam L, Gascon R, Stubblebine M, McGuire D, McGrath MS (1997). Unique monocyte subset in patients with AIDS dementia.. Lancet.

[7] Gartner S (2000). HIV infection and dementia.. Science.

[8] Ziegler-Heitbrock HW (1996). Heterogeneity of human blood monocytes: the CD14+ CD16+ subpopulation.. Immunol Today.

[9] Grage-Griebenow E, Flad HD, Ernst M (2001). Heterogeneity of human peripheral blood monocyte subsets.. J Leukoc Biol.

[10] Amirayan-Chevillard N, Tissot-Dupont H, Capo C, Brunet C, Dignat-George F (2000). Impact of highly active anti-retroviral therapy (HAART) on cytokine production and monocyte subsets in HIV-infected patients.. Clin Exp Immunol.

[11] Triques K, Stevenson M (2004). Characterization of restrictions to human immunodeficiency virus type 1 infection of monocytes.. J Virol.

[12] Almodovar S, Del CCM, Maldonado IM, Villafane R, Abreu S (2007). HIV-1 infection of monocytes is directly related to the success of HAART.. Virology.

[13] Zhu T, Muthui D, Holte S, Nickle D, Feng F (2002). Evidence for human immunodeficiency virus type 1 replication in vivo in CD14+ monocytes and its potential role as a source of virus in patients on highly active antiretroviral therapy.. J Virol.

[14] Crowe S, Zhu T, Muller WA (2003). The contribution of monocyte infection and trafficking to viral persistence, and maintenance of the viral reservoir in HIV infection.. J Leukoc Biol.

[15] Shiramizu B, Gartner S, Williams A, Shikuma C, Ratto-Kim S (2005). Circulating proviral HIV DNA and HIV-associated dementia.. Aids.

[16] Ellery PJ, Tippett E, Chiu YL, Paukovics G, Cameron PU (2007). The CD16+ monocyte subset is more permissive to infection and preferentially harbors HIV-1 in vivo.. J Immunol.

[17] Ancuta P, Kunstman KJ, Autissier P, Zaman T, Stone D (2006). CD16+ monocytes exposed to HIV promote highly efficient viral replication upon differentiation into macrophages and interaction with T cells.. Virology.

[18] Ancuta P, Autissier P, Wurcel A, Zaman T, Stone D (2006). CD16+ Monocyte-Derived Macrophages Activate Resting T Cells for HIV Infection by Producing CCR3 and CCR4 Ligands.. J Immunol.

[19] McArthur JC, Haughey N, Gartner S, Conant K, Pardo C (2003). Human immunodeficiency virus-associated dementia: an evolving disease.. J Neurovirol.

[20] Gonzalez-Scarano F, Martin-Garcia J (2005). The neuropathogenesis of AIDS.. Nat Rev Immunol.

[21] Kaul M, Garden GA, Lipton SA (2001). Pathways to neuronal injury and apoptosis in HIV-associated dementia.. Nature.

[22] Williams K, Westmoreland S, Greco J, Ratai E, Lentz M (2005). Magnetic resonance spectroscopy reveals that activated monocytes contribute to neuronal injury in SIV neuroAIDS.. J Clin Invest.

[23] McArthur JC (2004). HIV dementia: an evolving disease.. J Neuroimmunol.

[24] Cherner M, Cysique L, Heaton RK, Marcotte TD, Ellis RJ (2007). Neuropathologic confirmation of definitional criteria for human immunodeficiency virus-associated neurocognitive disorders.. J Neurovirol.

[25] Ancuta P, Rao R, Moses A, Mehle A, Shaw SK (2003). Fractalkine preferentially mediates arrest and migration of CD16+ monocytes.. J Exp Med.

[26] Douek DC, Brenchley JM, Betts MR, Ambrozak DR, Hill BJ (2002). HIV preferentially infects HIV-specific CD4+ T cells.. Nature.

[27] Gorry PR, Bristol G, Zack JA, Ritola K, Swanstrom R (2001). Macrophage tropism of human immunodeficiency virus type 1 isolates from brain and lymphoid tissues predicts neurotropism independent of coreceptor specificity.. J Virol.

[28] Ancuta P, Wang J, Gabuzda D (2006). CD16+ monocytes produce IL-6, CCL2, and matrix metalloproteinase-9 upon interaction with CX3CL1-expressing endothelial cells.. J Leukoc Biol.

[29] Kapadia F, Vlahov D, Donahoe RM, Friedland G (2005). The role of substance abuse in HIV disease progression: reconciling differences from laboratory and epidemiologic investigations.. Clin Infect Dis.

[30] Friedman H, Newton C, Klein TW (2003). Microbial infections, immunomodulation, and drugs of abuse.. Clin Microbiol Rev.

[31] Jirillo E, Caccavo D, Magrone T, Piccigallo E, Amati L (2002). The role of the liver in the response to LPS: experimental and clinical findings.. J Endotoxin Res.

[32] Dolganiuc A, Norkina O, Kodys K, Catalano D, Bakis G (2007). Viral and host factors induce macrophage activation and loss of toll-like receptor tolerance in chronic HCV infection.. Gastroenterology.

[33] Letendre S, Paulino AD, Rockenstein E, Adame A, Crews L (2007). Pathogenesis of hepatitis C virus coinfection in the brains of patients infected with HIV.. J Infect Dis.

[34] Parsons TD, Tucker KA, Hall CD, Robertson WT, Eron JJ (2006). Neurocognitive functioning and HAART in HIV and hepatitis C virus co-infection.. Aids.

[35] Morgello S (2005). The nervous system and hepatitis C virus.. Semin Liver Dis.

[36] Albillos A, de la Hera A, Gonzalez M, Moya JL, Calleja JL (2003). Increased lipopolysaccharide binding protein in cirrhotic patients with marked immune and hemodynamic derangement.. Hepatology.

[37] Opal SM, Scannon PJ, Vincent JL, White M, Carroll SF (1999). Relationship between plasma levels of lipopolysaccharide (LPS) and LPS-binding protein in patients with severe sepsis and septic shock.. J Infect Dis.

[38] Robertson KR, Smurzynski M, Parsons TD, Wu K, Bosch RJ (2007). The prevalence and incidence of neurocognitive impairment in the HAART era.. Aids.

[39] Brenchley JM, Schacker TW, Ruff LE, Price DA, Taylor JH (2004). CD4+ T cell depletion during all stages of HIV disease occurs predominantly in the gastrointestinal tract.. J Exp Med.

[40] Wright SD, Ramos RA, Tobias PS, Ulevitch RJ, Mathison JC (1990). CD14, a receptor for complexes of lipopolysaccharide (LPS) and LPS binding protein.. Science.

[41] Cohen IR, Norins LC (1966). Natural human antibodies to gram-negative bacteria: immunoglobulins G, A, and M.. Science.

[42] Titanji K, De Milito A, Cagigi A, Thorstensson R, Grutzmeier S (2006). Loss of memory B cells impairs maintenance of long-term serologic memory during HIV-1 infection.. Blood.

[43] Moir S, Malaspina A, Pickeral OK, Donoghue ET, Vasquez J (2004). Decreased survival of B cells of HIV-viremic patients mediated by altered expression of receptors of the TNF superfamily.. J Exp Med.

[44] Van Marle G, Rourke SB, Zhang K, Silva C, Ethier J (2002). HIV dementia patients exhibit reduced viral neutralization and increased envelope sequence diversity in blood and brain.. Aids.

[45] Ferrier L, Berard F, Debrauwer L, Chabo C, Langella P (2006). Impairment of the intestinal barrier by ethanol involves enteric microflora and mast cell activation in rodents Desamino-D-arg8-vasopressin (DDAVP), unlike ethanol, has no effect on a boring visual vigilance task in humans Thiopentone pharmacokinetics in patients with chronic alcoholism.. Am J Pathol.

[46] Caradonna L, Mastronardi ML, Magrone T, Cozzolongo R, Cuppone R (2002). Biological and clinical significance of endotoxemia in the course of hepatitis C virus infection.. Curr Pharm Des.

[47] Peng X, Cebra JJ, Adler MW, Meissler JJ, Cowan A (2001). Morphine inhibits mucosal antibody responses and TGF-beta mRNA in gut-associated lymphoid tissue following oral cholera toxin in mice.. J Immunol.

[48] Lucas GM, Griswold M, Gebo KA, Keruly J, Chaisson RE (2006). Illicit drug use and HIV-1 disease progression: a longitudinal study in the era of highly active antiretroviral therapy.. Am J Epidemiol.

[49] Hutchinson SJ, Brettle RP, Gore SM (1997). Predicting survival in AIDS: refining the model.. Qjm.

[50] Bouwman FH, Skolasky RL, Hes D, Selnes OA, Glass JD (1998). Variable progression of HIV-associated dementia.. Neurology.

[51] Tozzi V, Balestra P, Lorenzini P, Bellagamba R, Galgani S (2005). Prevalence and risk factors for human immunodeficiency virus-associated neurocognitive impairment, 1996 to 2002: results from an urban observational cohort.. J Neurovirol.

[52] Davidson DJ, Currie AJ, Bowdish DM, Brown KL, Rosenberger CM (2006). IRAK-4 mutation (Q293X): rapid detection and characterization of defective post-transcriptional TLR/IL-1R responses in human myeloid and non-myeloid cells.. J Immunol.

[53] Kobayashi K, Hernandez LD, Galan JE, Janeway CA, Medzhitov R (2002). IRAK-M is a negative regulator of Toll-like receptor signaling.. Cell.

[54] Chapoval AI, Kamdar SJ, Kremlev SG, Evans R (1998). CSF-1 (M-CSF) differentially sensitizes mononuclear phagocyte subpopulations to endotoxin in vivo: a potential pathway that regulates the severity of gram-negative infections.. J Leukoc Biol.

[55] Asakura E, Yamauchi T, Umemura A, Hanamura T, Tanabe T (1997). Intravenously administered macrophage colony-stimulating factor (M-CSF) specifically acts on the spleen, resulting in the increasing and activating spleen macrophages for cytokine production in mice.. Immunopharmacology.

[56] Rankine EL, Hughes PM, Botham MS, Perry VH, Felton LM (2006). Brain cytokine synthesis induced by an intraparenchymal injection of LPS is reduced in MCP-1-deficient mice prior to leucocyte recruitment.. Eur J Neurosci.

[57] Kusdra L, McGuire D, Pulliam L (2002). Changes in monocyte/macrophage neurotoxicity in the era of HAART: implications for HIV-associated dementia.. Aids.

[58] Gonzalez E, Rovin BH, Sen L, Cooke G, Dhanda R (2002). HIV-1 infection and AIDS dementia are influenced by a mutant MCP-1 allele linked to increased monocyte infiltration of tissues and MCP-1 levels.. Proc Natl Acad Sci U S A.

[59] Gartner S, Liu Y (2002). Insights into the role of immune activation in HIV neuropathogenesis.. J Neurovirol.

[60] Ryan LA, Zheng J, Brester M, Bohac D, Hahn F (2001). Plasma levels of soluble CD14 and tumor necrosis factor-alpha type II receptor correlate with cognitive dysfunction during human immunodeficiency virus type 1 infection.. J Infect Dis.

[61] Lien E, Aukrust P, Sundan A, Muller F, Froland SS (1998). Elevated levels of serum-soluble CD14 in human immunodeficiency virus type 1 (HIV-1) infection: correlation to disease progression and clinical events.. Blood.

[62] Zhou H, Lapointe BM, Clark SR, Zbytnuik L, Kubes P (2006). A requirement for microglial TLR4 in leukocyte recruitment into brain in response to lipopolysaccharide.. J Immunol.

[63] Xaio H, Banks WA, Niehoff ML, Morley JE (2001). Effect of LPS on the permeability of the blood-brain barrier to insulin.. Brain Res.

[64] Shen Y, Rudnik J, Cassol S, Drouin J, Cameron W (1994). Blood monocytes from most human immunodeficiency virus type 1-infected patients do not carry proviral DNA.. Clin Diagn Lab Immunol.

